# Casting light on the potential connection: exploring the relationship between periodic fever, aphthous stomatitis, pharyngitis and cervical adenitis (PFAPA) syndrome and Behҫet in the Druze population in Israel

**DOI:** 10.1186/s13023-026-04278-6

**Published:** 2026-04-24

**Authors:** Adan Gharra, Gil Amarilyo, Liora Harel, Yoel Levinsky, Rotem Tal, Adi Miller-Barmak, Bronya Sluvis, Yonatan Butbul Aviel

**Affiliations:** 1 The Ruth and Bruce Rappaport Faculty of Medicine, Technion-Israel Institute of Technology, Haifa, Israel; 2 Department of Pediatrics B, Ruth Rappaport Children’s Hospital, Rambam Medical Center, Haifa, Israel; 3 Pediatric Rheumatology Unit, Schneider Children’s Medical Center of Israel, Petach Tikva, Israel; 4 Sackler Faculty of Medicine, Tel Aviv University, Ramat Aviv, Israel; 5 Pediatric Rheumatology Service, Ruth Rappaport Children’s Hospital, Rambam Medical Center, Haifa, Israel

**Keywords:** Autoinflammatory disease, Behçet spectrum, Colchicine, Ethnic variation, MEFV gene, Periodic fever, PFAPA syndrome

## Abstract

**Objectives:**

To characterize the clinical presentation of PFAPA and the response to treatment in an ethnic subgroup- Druze and analyze the differences compared to other ethnic groups.

**Study design:**

Retrospective data were collected from medical records of patients with PFAPA attending 2 pediatric tertiary medical centers in Israel between March 2014-December 2022. Patients with concomitant familial Mediterranean fever were excluded. Ethnicity was categorized as Mediterranean, non-Mediterranean, multiethnic and Druze.

**Results:**

Of 386 patients with PFAPA, 52 (13.5%) were with a corresponding FMF diagnosis (PFAPA/FMF) information was lacking regarding FMF status in 9 (2.3%) patients and 8 (2.1%) patients were excluded because of poor follow-up. The study included 317 PFAPA patients, 178 (56.2%) of which were of Mediterranean descent (Sephardic Jews or Israeli Arabs), 87 (27.4%) were multiethnic, 19 (6%) were of non-Mediterranean descent (all Ashkenazi Jews), and 33 (10.4%) were of Druze ethnicity. No noteworthy differences in age of onset or age of diagnosis were found among ethnic groups. Clinical presentation analysis revealed statistical significance (*P* = 0.025) of the prevalence of abdominal pain across ethnic groups. No significance association was found regarding other symptoms including pharyngitis, adenitis, aphthous, myalgia, arthralgia, rash, and headache. Additionally, no significant association was observed between the response to treatment and ethnic group. Genetic testing was performed in 127 (40%) patients, no significant association was shown between number of mutations and ethnic origin.

**Conclusion:**

The clinical presentation of PFAPA in patients of Druze ethnicity is not significantly different from other ethnic groups in Israel. This finding fails to support the hypothesis that PFAPA is on the Behҫet spectrum.

**Supplementary Information:**

The online version contains supplementary material available at 10.1186/s13023-026-04278-6.

## Background

Periodic fever, aphthous stomatitis, pharyngitis, and adenitis (PFAPA) syndrome is the most common periodic fever condition in children [1]. It was first described by Marshall et al. in 1987 [2]. 

PFAPA syndrome is characterized by recurrent episodes of high fevers, along with the associated features of aphthous stomatitis, pharyngitis, and/or cervical adenitis [1, 3]. The genetic contribution as well as the pathogenesis of PFAPA are still unknown, although there is evidence that PFAPA is a disorder of innate immunity and responds to IL-1 blockade [4]. 

Familial clustering has been observed, suggesting that inherited genetic variants may contribute to risk [3–5]. To date, whole exome sequencing and linkage analyses have not identified any rare causative mutations linked to the disease, indicating that PFAPA is unlikely to follow a monogenic inheritance pattern in most patients [5]. Rather, PFAPA may reflect a complex genetic disorder.

In addition to PFAPA syndrome, aphthous ulcers and inflammation of the oropharyngeal mucosa are hallmarks of numerous other disorders such as Behҫet disease and recurrent aphthous stomatitis [6]. Based on gene and proteomic analyses and some clinical similarities, it has been suggested that PFAPA and Behҫet disease have similar disease mechanisms [7]. 

Behҫet disease is a systemic inflammatory disease primarily involving the oral and genital mucosa, skin, and eyes [8]. The most common initial manifestation of Behҫet disease is recurrent oral ulcers, between 63.8% and 98.2% in children [9–12]. The prevalence of Behҫet disease in the Druze population is in the range of 50–185: 100,000, which places the Druze among the populations with the highest prevalence all over the world [13]. 

PFAPA syndrome and Behҫet disease share similar clinical manifestations, such as oral ulcers, and both show a marked cytokine secretion distribution with IL-1b playing a key role in pathogenesis, although in different age groups [14]. Common genetic variants previously associated with Behҫet disease and recurrent aphthous stomatitis (another disease characterized by aphthous ulcers) are also strongly associated with PFAPA syndrome, the most significant being a variant upstream of IL12A [6]. 

PFAPA is increasingly considered a complex genetic disease involving multiple common genetic variants and environmental factors. Because aphthous ulcers frequently occur during PFAPA flares and are more prevalent among first-degree relatives of affected individuals, researchers have explored genetic overlap with recurrent aphthous ulcers and Behçet’s disease—both of which also feature mucosal inflammation [6]. 

Behçet’s disease is associated with both HLA (especially HLA-B*51:01) and non-HLA genetic risk variants identified through GWAS, including variants near **IL10**,** IL12A**,** STAT4**,** IL23R-IL12RB2**,** CCR1–3**,** TLR4**, and **TNFAIP3**, many of which affect immune pathways [15, 16]. Recurrent aphthous ulcers also show heritability (~ 8%) and share risk loci with Behçet’s disease, notably near **IL12A**,** IL10**, and **STAT4**, with variants in strong linkage disequilibrium across both conditions [17]. 

Given the phenotypic overlap, a study of 231 European-ancestry individuals with PFAPA assessed known risk variants from Behçet’s disease and recurrent aphthous ulcers. Significant associations were found near **IL12A** (OR = 2.13, *p* = 6 × 10⁻⁹), **IL10**, **STAT4**, and **CCR1–3**. Functional data showed that these variants influence cytokine expression: the **IL12A** variant increased IL-12 production, the **IL10** variant decreased IL-10 production, and the **STAT4** variant elevated STAT4 expression—suggesting enhanced Th1/Th17 immune responses during PFAPA flares [18, 19]. 

Additionally, both class I and class II **HLA haplotypes** were associated with PFAPA. The most significant was a class II haplotype (HLA-DQB1*06:03*,* HLA-DRB1*13:01, HLA-DQA1*01:03). While most HLA risk alleles were unique to PFAPA*, **HLA-B15:01** overlapped with recurrent aphthous ulcers and Behçet’s disease (in HLA-B*51-negative individuals) [20]. 

Due to the common clinical manifestations, genetic similarities and shared pathogenesis features, it was suggested that the three aphthous ulcers related disorders: recurrent aphthous stomatitis, PFAPA, and Behҫet disease, could be placed on a continuous spectrum and grouped as a family under the name Behҫet’s spectrum disorders (BCDs) with the mildest being recurrent aphthous stomatitis, PFAPA as the intermediary, and Behҫet disease as the most severe end of the spectrum [6]. 

The purpose of this study is to characterize the clinical presentation and the response to treatment in an ethnic subgroup- Druze and analyze the differences compared to non-Druze Arab population and the total PFAPA population. We Hypothesize that the Druze-subgroup has a different phenotype of PFAPA with features more similar to Behҫet disease. The aim is to assess the hypothesis that PFAPA is on the Behҫet spectrum by comparing the clinical features of PFAPA within the Druze population with high Behcet prevalence and other populations with PFAPA.

## Methods

### Study population

Retrospective data were collected and reviewed from children with a diagnosis of PFAPA who were treated at two major rheumatology centers in Israel: the pediatric rheumatology clinics at either Rambam’s Ruth Rappaport Children’s Hospital in Haifa, Israel or at Schneider’s Children’s hospital in Petah Tikvah, Israel, between March 2014- December 2022. Information regarding demography, ethnicity, diagnosis, follow-up, treatment, family history, genetics and more was collected at patient clinical visits and from medical records. Inclusion criteria for the study were clinical diagnosis of PFAPA by a pediatric rheumatologist and absence of other autoinflammatory manifestations. All tracked cases of PFAPA were validated according to previously established clinical criteria [21]. 

FMF is common in children of Mediterranean ancestry [22], therefore, to avoid referral bias, any patient with a concurrent diagnosis of FMF was excluded.

### Data collection

Patient ethnicity was categorized into Mediterranean, non-Mediterranean, multiethnic and Druze. Mediterranean ancestry was defined as ancestry of Mediterranean region of both parents, such as Sephardic Jews and Israeli Arabs. Non-Mediterranean are those with no ancestry of Mediterranean region on both sides. The Multiethnic group consists of children with one parent of Mediterranean ancestry and the other of non-Mediterranean ancestry. The Druze group, a subgroup of the Israeli Arabs, is a closed community which lives in Israel.

In addition to ethnic background, data were collected on clinical manifestations, frequency and duration of PFAPA flares, administration and response to various treatments such as corticosteroids, colchicine and tonsilloadenoidectomy, family history of PFAPA syndrome, recurrent pharyngitis, FMF and other autoimmune disease, family history of tonsillectomy, results of genetic tests, mainly FMF-related genes, which were ordered as part of the workup of periodic fever, either by the primary care physician or the pediatric rheumatologist. Response to corticosteroids was defined as defervescence within 12 h after intake of a single oral dose of corticosteroids. Response to colchicine was defined as a reduction or complete resolution of flares.

### Statistical analysis

All statistical analyses were performed using SPSS (version 26) and supplemented with post hoc power calculations in R. Descriptive statistics were used to summarize the data, with continuous variables presented as mean ± standard deviation (SD) and categorical variables as percentages. Group differences for categorical variables were assessed using the χ² test, with Fisher’s exact test applied when expected counts were less than 5 in 20% or more of the cells. Normality of continuous variables was evaluated using Shapiro-Wilk tests for each group. Outcome variables approximating normal distributions were analyzed using one-way ANOVA, with Levene’s test applied to assess homogeneity of variance. Post hoc comparisons were conducted using Tamhane’s T2 when the assumption of equal variance was violated, and Bonferroni correction was applied to adjust for multiple comparisons where appropriate. Effect sizes (η² and Cohen’s f) and power analyses were calculated using the harmonic mean of group sizes to account for unequal sample sizes (e.g., for Duration, Cohen’s f = 0.176, power = 0.28). Non-normal variables were analyzed using Kruskal-Wallis tests. The relatively low statistical power for some outcomes, such as Duration, reflects the unequal group sizes and sample limitations in this dataset. While these effect sizes provide useful estimates, the results should be interpreted with caution, and further studies with larger, more balanced cohorts are recommended to confirm these findings. A significance threshold of *P* < 0.05was applied for all analyses. This approach ensured robust and reliable statistical inference by appropriately balancing parametric and non-parametric methods, correcting for potential assumption violations, and accounting for multiple testing and statistical power.

## Results

### Study population

Of the 386 patients in the original database, 52 (13.5%) were excluded due to a concurrent diagnosis of FMF, 8 were excluded because of poor follow-up, and 9 (2.3%) were removed due to insufficient information regarding FMF status. In total, 317 patients were included in the study [See additional file 1].

Patients were stratified into ethnic groups: Druze, Ashkenazi, Mediterranean (includes Sephardic Jews and Arabs, Druze Arabs were excluded), and a mixed group. Table 1 summarizes the ethnic composition of the groups.

Statistical analysis was performed to compare the clinical characteristics of PFAPA syndrome between the different groups. Table 2 summarizes the demographic data of PFAPA patients by ethnic group. No noteworthy differences in age of onset were found among ethnic groups, in all groups there was an approximately 2-year delay until diagnosis. Regarding episode duration, variance heterogeneity was indicated by Levene’s test, and although ANOVA yielded a marginally significant result (*p* = 0.038), post hoc Tamhane’s T2 tests did not reveal specific group differences. There was no significant difference in the interval between episodes among ethnic groups.

With a p-value 0f 0.086, no association was found between a positive family history of FMF and ethnic origin although most of the FMF testing were done in mediterranean or mixed groups. In addition, no evidence was found to suggest a strong interconnection between ethnic origin with family history of any of the following: pharyngitis, tonsillectomy, PFAPA or other autoimmune disease.

Clinical symptoms that were compared between the groups included pharyngitis, adenitis, aphthous, abdominal pain, myalgia, arthralgia, rash, and headache. Table 3 summarizes the presence of clinical symptoms in patients with PFAPA by ethnic group The most common of which in all ethnic groups is pharyngitis, all the Druze patients included in this study (33) had a positive history of pharyngitis. The analysis of the presence of abdominal pain across ethnic groups was found to be significant. No significance was found in the analysis of the other symptoms.

**Table 1:** Distribution of study participants according to their ethnic origin group

Group	Origin	Frequency (*n*)	Percent (%)	
Ashkenazi group	Ashkenazi	19	6	
Mediterranean group	Sephardic	103	32.5	56.2
	Arab	75	23.7	
Mixed group	Mixed origin of Ashkenazi and Sephardic	87	27.4	
Druze group	Druze	33	10.4	
Total		317	100	

**Table 2:** Clinical and demographic characteristics of patients according to ethnic origin group

Variable	Ashkenazi (*n* = 19)	Mediterranean (*n* = 178)	Multiethnic (*n* = 87)	Druze (*n* = 33)	*P* value
Age of onset (y), mean ± SD	3.78 ± 1.8	2.87 ± 1.98	3.21 ± 2.03	2.4 ± 1.7	0.07
Age of diagnosis (y), mean ± SD	6.2 ± 2.7	4.9 ± 2.5	5.04 ± 2.4	4.7 ± 2.2	0.46
Duration of episode (days), mean ± SD	5.11 ± 2.2	4.52 ± 1.2	3.97 ± 1.6	3.95 ± 1.5	Not significant
Interval between episodes (weeks), mean ± SD	4.74 ± 3.4	3.8 ± 2.2	4.03 ± 2.4	3.5 ± 1.6	0.265
FH of FMF, n/N (%)	0	41/176 (23.3%)	15/86 (17.4%)	8/33 (24.2%)	0.086
FH of PFAPA, n/N (%)	1/19 (5.3%)	33/173 (19.1%)	11/86 (12.8%)	4/33 (12.1%)	0.274
FH of autoimmune disease, n/N (%)	3/19 (15.8%)	38/174 (21.8%)	22/86 (25.6%)	5/33 (15.1%)	0.586
FH of pharyngitis, n/N (%)	4/19 (21%)	42/173 (24.3%)	20/85 (23.5%)	7/33 (21.2%)	0.974
FH of tonsillectomy, n/N (%)	4/10 (40%)	33/118 (28%)	12/39 (30.8%)	13/32 (40.6%)	0.533

FH=Family History

**Table 3:** Frequency of clinical symptoms among patients according to ethnic origin group

Symptoms, %	Ashkenazi	Mediterranean	mixed	Druze	Total	*P* value
Pharyngitis	94.74%	94.35%	90.7%	100%	93.97%	0.275
Adenitis	47.37%	49.72%	52.33%	69.7%	52.38%	0.198
Aphthous	36.84%	36.72%	31.4%	50%	36.62%	0.327
Abdominal pain	26.32%	53.67%	55.81%	69.7%	54.29%	**0.025**
Myalgia	36.84%	24.29%	33.72%	28.12%	28.02%	0.345
Arthralgia	36.84%	24.29%	33.72%	28.12%	28.02%	0.345
Rash	0%	5.65%	5.81%	9.09%	5.71%	0.686
Headache	10.53%	15.34%	16.28%	12.5%	15.02%	0.937

### MEFV gene mutations across ethnic groups

127 patients underwent genetic testing for common MEFV mutations. The patients were categorized into groups based on the number of mutations found: no mutations, one mutation and more than one mutation. Table 4 summarizes the prevalence in each genetic category by ethnic origin. In 4 patients the testing results were unknown. No significant association was shown between number of mutations and ethnic origin (P value = 0.718).

The most common mutation found was heterozygote M694V, it was found in 31 (24.4%) patients. The second and third were heterozygote E148Q and V726A found in 14 (11%) and 9 (7%) patients respectively.

**Table 4:** Distribution of MEFV gene mutations among patients by ethnic origin group

Mutations, *n*	Ashkenazi	Mediterranean	Mixed	Druze	Total
No mutations	2	34	15	9	60
One mutation	1	39	13	5	58
More than one	0	4	0	1	5
Unknown	0	2	1	1	4
Total	3	79	29	16	127

### Treatment and response to therapy across ethnic groups

Treatment administered and response to treatment compared and analyzed between the groups. 93.8% of patients received treatment with corticosteroid at flares. About 90% of those who reported back after receiving treatment described symptomatic relief within a few hours of the initial dose. The cross-tabulation analysis for the treatment response episode resolving with steroids across origin groups did not reveal a significant association. 97 (30.6%) patients received prophylactic treatment with colchicine. Information regarding response to colchicine treatment was available on 75 patients, 54 (72%) of which reported reduction of flare frequency with treatment. No significant association was found between response to colchicine and ethnic origin (P value = 0.052). Other treatments available were cimetidine, Montelukast and adenotonsillectomy, statistical analysis was not performed regarding response to those treatments due to the small number of patients.

## Discussion

Multiple studies suggested a connection between PFAPA syndrome and Behҫet disease [6, 14, 23], we attempted to detect a difference in clinical presentation of PFAPA syndrome in an ethnic group with predisposition to Behҫet disease.

In this study we did not find a significant difference in the clinical presentation of PFAPA syndrome between ethnic groups from two tertiary Israeli hospitals. The study included 317 PFAPA patients, 178 (56.2%) of which were of Mediterranean descent (Sephardic Jews or Israeli Arabs), 87 (27.4%) were multiethnic, 19 (6%) were of non-Mediterranean descent (all Ashkenazi Jews), and 33 (10.4%) were of Druze ethnicity. Abdominal pain was the only symptom to yield statistical significance across ethnic groups (p-value = 0.025). Abdominal pain during attacks was reported by 69.9% of the Druze group, 53.67% and 55.81% of the Mediterranean and mixed group, compared with 26.32% of the Ashkenazi group. Another finding worth mentioning is the response to colchicine treatment- even though not statistically significant, the proximity of the P value (= 0.052) to the statistical threshold suggests a potential association between origin group and response to treatment with colchicine.

Other components of clinical presentation including age of onset, age of diagnosis, pharyngitis, adenitis, aphthous stomatitis failed to achieve statistical significance. In addition, the number of MEFV mutations and response to treatments was similar across ethnic groups.

The average population of Israel composes of approximately 78.74% Jews and 21.26% Arabs. Among Arabs 84.2% are Muslims, 8.6% are Christians and 7.2% are Druze [24]. About 150,000 Druze reside in Israel, mostly in the northern district of the country [24]. The Druze communities in Israel are clustered in three major regions: the Carmel Mountain, the Upper Galilee, and the Golan Heights [25]. For centuries, the Druze population have strictly prohibited marriage to non-Druze and limited conversion into the religion. In addition, Druze typically marry within the extended family resulting in a high rate of (47%) consanguineous marriages and residence in isolated regions [25, 26]. This combination makes the Druze community unique and fit for genetic research [25, 26]. As mentioned previously, The prevalence of Behҫet disease in the Druze population is in the range of 50–185: 100,000, which places the Druze among the populations with the highest prevalence all over the world [13]. 

Manthiram et al. proposed a theory suggesting a connection between PFAPA syndrome and Behҫet disease placing them on a common spectrum with recurrent aphthous stomatitis with Behҫet disease on the severe end, recurrent aphthous stomatitis on the mild end, and PFAPA intermediate to which the authors gave the name of Behcet spectrum disorders. This theory was based on clinical and genetic similarities among these disorders. It suggested that these conditions have similar disease mechanisms and the disease form developed depended on differing environmental and host factors (mainly in the form of different HLA associations). The study included 231 patients with PFAPA from three different cohorts and screened them for variants previously associated with Behcet disease or recurrent aphthous stomatitis. A strong association was found between PFAPA and the common variants previously mentioned [6]. 

In another study which included 80 adult patients with definite Behҫet diagnosis from 7.5% up to 30% of patients with Behҫet disease fulfilled PFAPA diagnostic criteria, depending on the specialty of the doctor taking the history. Although during childhood most of the patients suffered from recurrent aphthous stomatitis in childhood, no patients fulfilled Behҫet diagnostic criteria. Therefore, Cantarini et al. suggest a common immune imbalance underlying both PFAPA and Behҫet disease. The number of years between resolution of PFAPA signs and the onset of Behҫet disease was unclear [14]. 

In a follow-up study of 83 PFAPA patients, the affected children had no long-term sequelae. The follow up ranged from 12 to 21 years. The average age of the subjects was 20.34 ± 4.25 years. Behҫet disease was diagnosed in one patient at the age of 14. The possibility of early mouth ulcers of Behҫet being mistaken for PFAPA was raised. However, her initial presentation was characteristic of PFAPA suggesting relationship between these two conditions. A few patients were diagnosed with other conditions, and since the pathogenesis of the disease remains unclear, Wurster et al. suggest that PFAPA may represent a spectrum of disease with varied etiology [23]. 

The age of onset of PFAPA syndrome is below 5 years of age [3], while Behҫet disease occurs mainly between 18 and 40 years of age [9]. Since it was formerly observed that no Behҫet patient fulfilled the Behҫet diagnostic criteria during childhood [14] and in another research with a follow-up of up to 21 years only one patient developed Behҫet disease [23], a longer follow up period or a reverse study design might have yielded more significant results.

In several studies abdominal pain was reported in 16.4–86.3% of PFAPA patients [27–33]. In a previous study by our group abdominal pain was present in 90% of patients [4] and in our current one it was present in 54.3%. The statistically significant difference found in our study between groups is likely due to lower prevalence of abdominal pain in the Ashkenazi group (26.3%) compared to the other groups yet still within previously reported range. However, since the non-Ashkenazi group consists only of 19 patients, no definitive conclusions can be drawn.

In our study, Colchicine was prescribed as prophylactic regimen in PFAPA with reported efficacy of 40–60% [4, 34]. Colchicine is also a known and effective prophylactic regimen in FMF with an approximate efficacy of 90% [35]. Overall, 97 patients received prophylactic Colchicine treatment, data regarding response was available on 75 patients. The Ashkenazi group consisted of one patient; therefore, no conclusions can be drawn about response to colchicine in this ethnic group. The response rate to colchicine was 80.4%, 52.9% and 50% in the Mediterranean, Non-mediterranean and Druze groups respectively. The difference in colchicine response rate between the groups was close to statistical significance (P value = 0.052). A response rate of 80.4% in the Mediterranean group is higher than the reported rates found in previous studies of around 50% in PFAPA patients [4, 34]. The higher response rate in the Mediterranean group could be explained by higher carrier rates of MEFV variants in patients of Mediterranean ancestry reported in literature [22]. In our study we cannot confidently compare carriage rates of MEFV mutation among ethnic groups because only three patients of non-Mediterranean origin were screened in addition to the exclusion of patient with a concurrent diagnosis of FMF.

This study had a few limitations. The retrospective design constructed an ingrained bias. The study was performed on patients attending two tertiary hospitals; therefore, it is likely that they present with a more severe phenotype, whilst patients with a milder presentation are most probably treated by primary care physicians and smaller health centers, resulting is a severity-related referral bias, consequently our findings are not to be generalized to the whole Israeli population. In addition, the Druze and the Ashkenazi groups were small compared to other groups (33 and 19 patients, respectively), which made the comparison and achieving statistical significance challenging. Moreover, this study relied on retrospective clinical diagnoses of PFAPA made by pediatric rheumatologists according to established criteria; however, no universally accepted diagnostic test for PFAPA exists. The differentiation between PFAPA, early-onset Behçet disease, and other autoinflammatory syndromes can be challenging, particularly in populations with high Behçet prevalence such as the Druze [36]. Because laboratory or genetic biomarkers specific to PFAPA are lacking, diagnostic overlap or misclassification cannot be fully excluded. Additionally, since FMF and other hereditary periodic fever syndromes may share clinical features with PFAPA, even though patients with confirmed FMF were excluded, some degree of undetected overlap may persist. These diagnostic limitations may have influenced the observed homogeneity between ethnic groups and could partially account for the absence of significant differences in clinical presentation.

In conclusion, this study showed no noteworthy differences in the clinical presentation of PFAPA syndrome in the Druze population compared to other ethnic groups in Israel. Given the theory of Behҫet’s spectrum disorders (BCDs) which places PFAPA syndrome on the continuous spectrum with Behҫet disease and recurrent aphthous stomatitis, with Behҫet disease as the most severe end of the spectrum [6], our hypothesis assumed that the clinical presentation of PFAPA syndrome in the Druze population, a population with a significantly higher prevalence of Behҫet disease, would be more severe compared to other ethnic groups and more similar to the clinical presentation of Behҫet disease. Admittedly, the results were divergent, the clinical presentation including age of onset, episode duration and prevalence of aphthous stomatitis showed no statistical significance compared to other ethnic groups. The only symptom achieving statistical significance was abdominal pain being highest among Druze patients (69.7%) making the second most reported symptom together with adenitis (69.7%) after pharyngitis (100%). We note that abdominal pain is in second place in the Mediterranean and mixed group as well. Our findings fail to support the theory of Behҫet’s spectrum disorders.

## Conclusion

In this study, we found no significant differences in the clinical presentation or treatment response of PFAPA syndrome among Druze patients compared to other ethnic groups in Israel. Despite the Druze population’s high prevalence of Behçet disease, our findings do not support a clinical overlap between PFAPA and Behçet within this group. These results suggest that PFAPA in Druze patients does not represent a distinct phenotype and challenge the hypothesis that PFAPA lies on the Behçet disease spectrum. Further large-scale, prospective, and genetic studies are warranted to clarify the shared and divergent pathogenic mechanisms of these autoinflammatory conditions.

## Supplementary Information

Below is the link to the electronic supplementary material.

Supplementary Material 1

## Data Availability

The dataset supporting the conclusions of this article is included within the article and its additional file. “Additional file 1” is an excel format file (.xlsx) which contains the complete, anonymized dataset used in this study.

## Abbreviations

PFAPA: Periodic Fever, Aphthous Stomatitis, Pharyngitis, and Adenitis
FMF: Familial Mediterranean Fever
BCDs: Behçet’s Spectrum Disorders
CAPS: Cryopyrin-Associated Periodic Syndromes
GWAS: Genome-Wide Association Studies
